# Rare Colonic Dieulafoy Lesions: A Case Series

**DOI:** 10.7759/cureus.62446

**Published:** 2024-06-15

**Authors:** Nancy Jhanji, Connor Lovingood, William K Oelsner, James E Pitcher, Laxmi Parsa

**Affiliations:** 1 Internal Medicine, University of Tennessee College of Medicine, Chattanooga, USA; 2 Gastroenterology, University of Tennessee College of Medicine, Chattanooga, USA; 3 Gastroenterology and Hepatology, University of Tennessee College of Medicine, Chattanooga, USA

**Keywords:** high-risk bleeds, esophagogastroduodenoscopy (egd), colonoscopy, gastrointestinal bleeding (gib), hemorrhagic shock, colonic dieulafoy, case report, gi bleeds, dieulafoy lesions

## Abstract

Dieulafoy lesions (DLs) are infrequent causes of gastrointestinal bleeding (GIB) but can cause hemorrhage with a high risk of re-bleeds. They are most noted in the stomach, but this case series of three colonic DLs highlights even more rare causes of lower GIB.

Three patients presented with blood loss and were found to have colonic DLs. All of them had esophagogastroduodenoscopies (EGDs) that were unremarkable, and they subsequently underwent a colonoscopy, which then showed oozing DLs. First, a 63-year-old woman had a week of maroon-colored stools but no use of blood thinners, prior GIB, or peptic ulcers. Next, an 81-year-old man presented with dyspnea and had a two-week history of melena. Three years later, he presented with two oozing lesions on a colonoscopy, which likely indicated a repeat DL. This was followed by multiple admissions for GIB. The lesions in these two cases were treated with epinephrine and hemostatic clips. Lastly, a 49-year-old man presented with hematochezia leading to shock, requiring transfusions, vasopressors, and ICU care. Computed tomography angiography (CTA) showed intraluminal contrast extraversion in the ascending colon, leading to interventional radiology (IR)-guided coil for suspected DL.

Diagnosis can be hard, but early identification through endoscopy can help decrease mortality rates. Therefore, it is crucial to keep this on the list of differential diagnoses in cases with no other identifiable sources to allow for timely management.

## Introduction

Dieulafoy lesions (DLs) are a type of vascular lesions that may cause bleeding in the gastrointestinal (GI) tract. They are dilated submucosal vessels that erode into the mucosa without obvious signs of surrounding ulcers, aneurysms, or mucosal abnormalities [1,2]. They account for 1%-6% of all causes of acute upper gastrointestinal bleeding (GIB) [3,4]. The following cases discuss the presentation of exceedingly rare colonic DLs, which account for 0.09% of lower GIB [5]. Given the low incidence, it is important to consider it as part of a differential diagnosis in hemodynamically unstable patients without other identifiable sources of GIB and be aware of treatment options. They are twice as common in males as compared to females [5]. The majority of lesions are found in the stomach (70%), especially on the lesser curvature within 6 cm of the gastroesophageal junction [1,6]. Here, we present a case series in which three patients with lower GIBs were found to have colonic DLs.

## Case presentation

**Figure 1:**
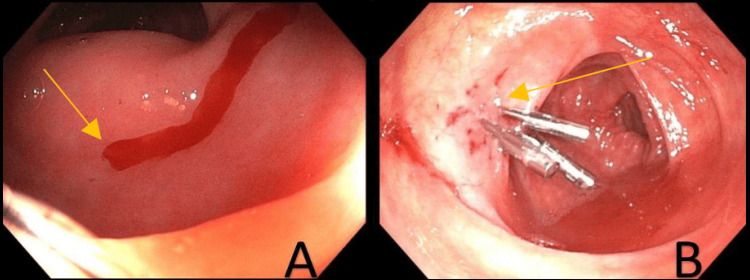
Figure 1A shows this patient's active oozing Dieulafoy lesion. Figure 1B shows treatment using epinephrine and three clips.

The first patient was a 63-year-old South Asian female with hypertension, type 2 diabetes mellitus, hypothyroidism, and severe aortic stenosis who presented to the hospital for an outpatient coronary angiogram in anticipation of AV repair. Incidentally, she was found to have a hemoglobin of 6.4 g/dL (normal range: 12-16 g/dL) from her baseline of 13 g/dL and was admitted for evaluation of a GI bleed. Her other labs were unremarkable, and she reported one week of maroon-colored stools. She was not on any blood thinners and denied any previous history of GIB or peptic ulcers. Her last colonoscopy, seven years ago, was normal. An esophagogastroduodenoscopy (EGD) performed showed Los Angeles (LA) grade A esophagitis and acute duodenitis without a clear source of acute blood loss. The colonoscopy showed non-bleeding hemorrhoids, as well as bright red blood, present up to the proximal transverse colon, where two bleeding DLs were noted distal to the hepatic flexure (Figure 1A). There were no surrounding mucosal abnormalities or ulcerations. The area was successfully injected with epinephrine, and three clips were placed (Figure 1B). There was no further active bleeding seen during the hospitalization or at outpatient follow-ups.

**Figure 2:**
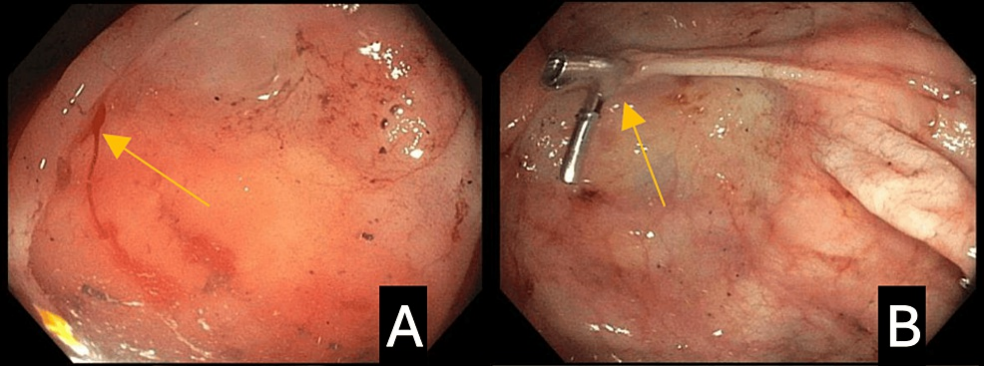
Figure 2A shows the Dieulafoy lesion in the cecum with retrograde flow into the terminal ileum. Figure 2B shows the lesion after it was treated with epinephrine and two hemostatic clips.

The second case involved an 81-year-old White male patient with atrial fibrillation on apixaban, congestive heart failure with an ejection fraction (EF) of 40%-45%, prior myocardial infarction with percutaneous intervention and bare metal stenting, hypertension, iron deficiency anemia, and mild dementia who presented with dyspnea and chest pain secondary to demand ischemia from acute blood loss anemia. His initial hemoglobin was 5.6 g/dL (normal range: 12-16 g/dL), which responded appropriately to a blood transfusion. He endorsed a two-week history of dark-colored stools, which had never occurred in the past. He was up-to-date on his screening colonoscopies. His EGD showed normal mucosa throughout; however, a colonoscopy revealed blood within the entirety of the colon with the identification of DL in the cecum with the retrograde flow into the terminal ileum (Figure 2A). The lesion was injected with epinephrine, followed by the placement of two hemostatic clips (Figure 2B). A subsequent video capsule endoscopy was unrevealing. No further complications were noted at subsequent office visits for this encounter. Three years later, the patient presented to the emergency department once again with acute blood loss and anemia secondary to GIB. The patient did not require transfusion, and a colonoscopy revealed two non-specific areas of abnormal mucosa in the cecum with actively oozing blood, requiring hemostatic clipping for source control, likely representing a repeat DL bleed. Unfortunately, he returned a year later for a lower GI bleed from an arteriovenous (AV) malformation, followed by two more admissions for melena, where no active bleeding was identified.

**Figure 3:**
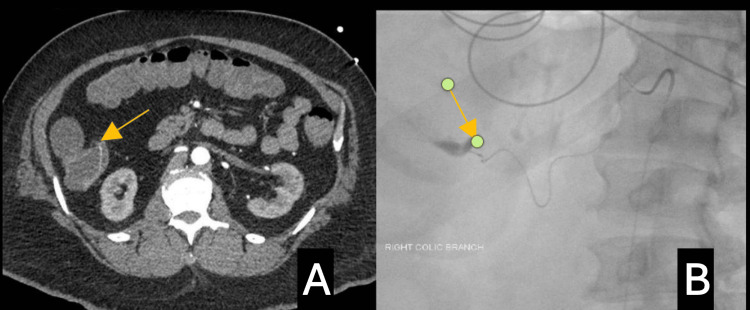
A CT angiogram of the abdomen shows contrast extraversion within the ascending colon (Figure 3A). Interventional radiology (IR)-coil embolization of the right colic artery with a suspected Dieulafoy lesion is seen (Figure 3B).

The third patient was a 49-year-old male with a medical history of hypertension, type 2 diabetes mellitus, gout, and a prior cerebral vascular accident (CVA) who presented with four episodes of bright red blood per rectum along with lightheadedness and diaphoresis. He had a colonoscopy done that same year, notable for internal hemorrhoids and polyps. He was taking aspirin (81 mg) daily at home. He continued to have multiple episodes of active bleeding in the ER, leading to hemorrhagic shock and requiring blood transfusions and vasopressors. He was transferred to the ICU, where bedside EGD did not reveal a source of bleeding, and the colonoscopy showed blood throughout the colon with diverticula in the transverse, descending, and sigmoid colon. No active bleeding was found during the exam. After the procedure, the patient recovered and underwent a CT angiogram, which showed intraluminal contrast extraversion within the ascending colon (Figure 3A). He required interventional radiology (IR)-guided coil embolization of the right colic submucosal-appearing artery suspected to be a DL case (Figure 3B). The patient was discharged and seen in the clinic a month later without any episodes of rebleeding.

## Discussion

Dieulafoy lesions are unique in that they tend to maintain their large caliber despite being distal in the submucosa, unlike other arteries that tend to narrow [4,5]. When they protrude into the mucosa, they are at risk of being further exposed to the lumen, which can then lead to perforation and active bleeding from any minor trauma. Affected patients generally have a prior history of cardiovascular disease, hypertension, diabetes, chronic kidney disease, and anticoagulation use [4].

These lesions can occur at any age but are more likely to be seen in the sixth or seventh decades of life, which could be related to long-term age-related mucosal thinning [5]. This suggests these lesions are likely acquired defects; however, there is little data to support this hypothesis [5]. The diagnosis can be challenging due to the presence of diverticula, bleeding cessation prior to diagnostic endoscopy, and difficulty locating the lesion in the setting of poor bowel prep [7-9]. The lesions present with acute, painless, severe hematemesis, melena, and/or hematochezia, with 51% presenting with hematemesis and melena. Colonic DLs generally have been reported to have significant bright red blood per rectum. The absence of abdominal pain may help distinguish this diagnosis from other causes of lower GIB, such as mesenteric ischemic and peptic ulcer disease in upper GIB or diverticulitis, hemorrhoids, and inflammatory bowel disease [4].

The initial diagnostic test is an EGD, where 50%-60% of the time lesions can be seen actively spurting or oozing from a source [5,8]. Other cases noted in the literature have had instances many times where a spurting lesion was not pinpointed, just like in the third patient in this series; however, in patient one, the lesion was luckily identified during the first endoscopy. However, in the second patient's case, not every EGD during repeat admissions for rebleeding was successfully able to capture actively oozing lesions. Various cases reported previously have suspected the source to be a DL due to the diagnosis of exclusion due to no direct visualization. Therefore, this case series and figures are unique in that they highlight three different diagnostic findings that colonic DLs can present with. Overall, this can contribute to a better understanding of what to look for to get a timely diagnosis for patients. Often, a clot can cover the lesion, making it challenging to identify the source [8]. If certain clots are noted, it is not advised to uncover them due to the risk of severe hemorrhage [5]. It is important to be able to distinguish bleeding lesions from vascular neoplasms, telangiectasias, arteriovenous malformations, superficial mucosal tears, or adenomatous polyps [5]. Being certain of what type of lesion is present is crucial for appropriate treatment. For example, DLs mistakenly believed to be polyps can hemorrhage if a polypectomy is attempted [5].

Dieulafoy lesions can generally be diagnosed if there is active spurting, the presence of a clot, or a protruding vessel with a small mucosal defect [8]. A non-diagnostic EGD is followed up by a colonoscopy and later an enteroscopy. Some DLs can be identified by a pill cam [5]. In cases of active bleeding and unremarkable endoscopies, angiography can be the next step for diagnosis [5]. Initial management for affected patients involves hemodynamic stability with volume resuscitation and blood transfusions. The treatment modalities of the past included surgical intervention; however, the standard therapy now is therapeutic endoscopy followed by therapeutic angiography as a second-line option [6, 7]. Certain patients can be candidates for therapeutic angiography if the bleeding site is inaccessible to endoscopy or if patients are poor surgical candidates [4].

These advancements in endoscopic interventions have decreased mortality from 80% to 8.6% due to improved early detection [7]. The most common methods of endoscopic treatment include injections, ablation, or mechanical therapy [5]. Injections with epinephrine are commonly used but are limited in patients with arrhythmias, hemodynamic instability, and recent myocardial infarction. Other injectables include sclerosing agents, which result in local irritation leading to inflammation and thrombosis, as well as cyanoacrylate, which glues the bleeding vessel [5]. Ablation therapy involves argon plasma coagulation (APC), thermocoagulation, and electrocoagulation. Argon plasma coagulation differs from the other two methods in that contact with the lesion is not required [5]. Finally, mechanical therapy involves the placement of endoscopic clips or ligation. A combination of endoscopic interventions can be used and may be more effective in patients who are at higher bleeding risk [4].

## Conclusions

Although rare, DLs can cause active, heavy GIB that can lead to hemorrhagic shock, and if left untreated, bleeding can frequently reoccur less than 72 hours after the initial presentation. With advancements in endoscopic procedures, the prevalence of these lesions has increased; therefore, it is crucial to include DLs as part of a differential diagnosis, especially in cases where no additional clear sources are discovered to allow for appropriate and timely management.
